# Nobiletin Inhibits Hepatic Lipogenesis via Activation of AMP-Activated Protein Kinase

**DOI:** 10.1155/2018/7420265

**Published:** 2018-02-07

**Authors:** Taewon Yuk, Younghwa Kim, Jinwoo Yang, Jeehye Sung, Heon Sang Jeong, Junsoo Lee

**Affiliations:** ^1^Division of Food and Animal Sciences, Chungbuk National University, Cheongju, Chungbuk 28644, Republic of Korea; ^2^School of Food Biotechnology and Nutrition, Kyungsung University, Busan 48434, Republic of Korea

## Abstract

We aimed to investigate the effects of nobiletin on hepatic lipogenesis in high glucose-induced lipid accumulation in HepG2 cells. Nobiletin, a citrus polymethoxyflavonoid with six methoxy groups, is present abundantly in the peels of citrus fruits. HepG2 cells were incubated in Dulbecco's modified Eagle's medium containing high glucose (25 mM) and subsequently treated with nobiletin at different concentrations (5, 25, and 50 *μ*M). Results showed that nobiletin markedly inhibited high glucose-induced hepatic lipid accumulation in HepG2 cells. In addition, it reduced the protein expression of lipogenic factors, including sterol regulatory element-binding protein 1c (SREBP-1c) and fatty acid synthase (FAS). Nobiletin significantly increased the phosphorylation of AMP-activated protein kinase (AMPK) and acetyl-CoA carboxylase. Pretreatment with compound C, an AMPK inhibitor, abolished the inhibitory effects of nobiletin on SREBP-1c and FAS expression. These results suggested that nobiletin might attenuate high glucose-induced lipid accumulation in HepG2 hepatocytes via modulation of AMPK signaling pathway. Therefore, nobiletin might be useful for the prevention and treatment of nonalcoholic fatty liver diseases.

## 1. Introduction

The liver plays a key role in lipid metabolism. It is well known that a high‐carbohydrate diet can prime the hepatic de novo lipogenesis (DNL) pathway with a large substrate load and, thus, increase the rate of DNL [1]. Accumulation of lipid droplets in the hepatocytes has been closely related to obesity, insulin resistance, type 2 diabetes, and nonalcoholic fatty liver diseases (NAFLD) [2, 3]. Elevated hepatic DNL was observed in insulin resistance condition [4–6]; therefore, a finding of high DNL rate in an individual may provide early warning of the possible development of type 2 diabetes mellitus. Therefore, the prevention of increased DNL in the liver may provide a therapeutic strategy for the management of multiple stages of NAFLD.

Hepatic AMP-activated protein kinase (AMPK) is thought to play a pivotal role in regulating lipid metabolism, glucose homeostasis, and insulin sensitivity [7, 8]. As an energy sensor maintaining cellular glucose homeostasis, AMPK significantly inhibits hepatic glucose output by transcriptional control [9]. In addition, AMPK regulates acyl coenzyme A (acyl-CoA) channeling towards *β*-oxidation and away from glycerolipid biosynthesis. AMPK phosphorylates and downregulates acetyl-CoA carboxylase (ACC), thereby decreasing the production of malonyl-CoA. Sterol regulatory element-binding proteins (SREBPs) are key lipogenic transcription factors that regulate the expression of lipogenic enzymes, including ACC, fatty acid synthase (FAS), and 3-hydroxy-3-methylglutaryl-CoA reductase [10–12]. Overexpression of hepatic SREBP-1c results in selective induction of lipogenic gene expression, with no effect on the genes involved in cholesterol synthesis [13]. Moreover, the phosphorylation of AMPK inactivates SREBP-1 and inhibits hepatic lipid accumulation in a high-fat diet-induced mouse [14, 15].

Nobiletin, a major component in citrus fruits [16], has been used in traditional Chinese herbal medicine [17]. It has been shown to possess several biological activities, including anti-inflammatory [18], antitumor [19], antidiabetic [20], and antiobesity [21] effects, as well as play an important role in the prevention of postprandial hyperglycemia [22] and hyperlipidemia [23]. However, the molecular mechanisms underlying its effects on hepatic lipid metabolism, particularly with regard to the activation of AMPK, are still unclear. In this study, we investigated the antilipogenic effects of nobiletin on high glucose-induced lipid accumulation in HepG2 cells through the AMPK signaling pathway.

## 2. Materials and Methods

### 2.1. Chemicals

**Figure 1:**
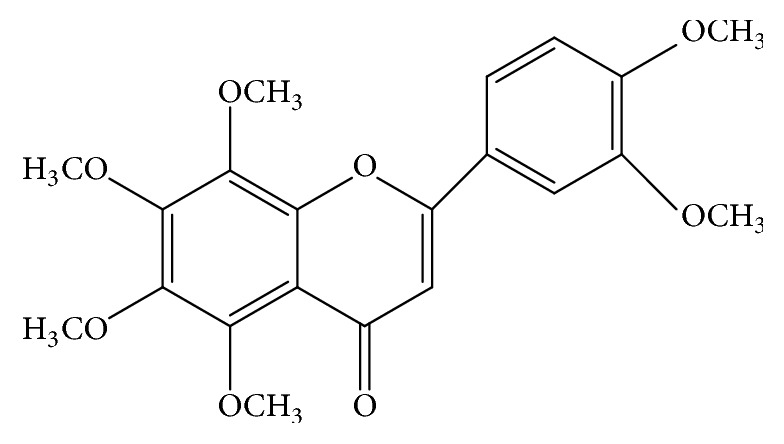
Structure of nobiletin.

Nobiletin (purity ≥ 95%) was purchased from Wako Pure Chemicals (Osaka, Japan) (Figure 1). Dulbecco's modified Eagle's medium (DMEM), fetal bovine serum (FBS), trypsin-ethylenediaminetetraacetic acid (EDTA), and penicillin-streptomycin were obtained from Gibco BRL (Grand Island, NY, USA). 6-[4-(2-Piperidin-1-ylethoxy)phenyl]-3-pyridin-4-ylpyrazolo[1,5-a]pyrimidine (Compound C), 3-(4,5-dimethylthiazol-2-yl)-2,5-diphenyltetrazolium (MTT), dimethyl sulfoxide (DMSO), lactate dehydrogenase (LDH) kit, and Oil red O (ORO) were purchased from Sigma-Aldrich (St. Louis, MO, USA). Antibodies against p-AMPK, p-ACC, AMPK, and ACC were obtained from Cell Signaling Technology (Beverly, MA, USA). Antibodies against SREBP-1c, FAS, and *β*-actin were purchased from Santa Cruz Biotechnology (Santa Cruz, CA, USA). Enhanced chemiluminescence (ECL™) detection reagents were purchased from GE Healthcare (Buckinghamshire, UK). All other reagents and solvents were of analytical and HPLC grades.

### 2.2. HepG2 Cell Culture and Hepatic Lipogenesis

HepG2 cells were obtained from the Korean Collection for Type Cultures (KCTC, Daejeon, Korea) and cultured in DMEM supplemented with 10% heat-inactivated FBS, 100 U/mL penicillin, 100 *μ*g/mL streptomycin, and 5.5 mM D-glucose. HepG2 cells were maintained in a humidified incubator with 5% CO_2_ at 37°C.

A cell model of high glucose-induced lipid accumulation in hepatocytes was prepared by exposing HepG2 cells to a high concentration of glucose (25 mM) for 24 h, as previously described [24]. Briefly, HepG2 cells were cultured in FBS-free medium overnight to induce serum depletion. Then, HepG2 cells were incubated in FBS-free medium containing high concentration of glucose (25 mM) and subsequently treated with nobiletin at different concentrations (5, 25, and 50 *μ*M).

### 2.3. Cytotoxicity

After 24 h, MTT reagent (0.5 mg/mL) dissolved in FBS-free DMEM was added to each well, and the cells were incubated for 3 h at 37°C. The culture medium was removed, and the intracellular formazan product was dissolved by adding DMSO. The absorbance was measured at 550 nm using a spectrophotometer (BioTek Instruments, Inc., Winooski, VT, USA).

### 2.4. Oil Red O Staining

To measure intracellular lipid accumulation, HepG2 cells were stained by ORO. HepG2 cells were cultured in FBS-free medium overnight to induce serum depletion. After 24 h, HepG2 cells were incubated in FBS-free medium containing either normal (5.5 mM) or high (25 mM) concentration of D-glucose and subsequently treated with nobiletin at different concentrations. After treatment, HepG2 cells were washed twice with cold phosphate-buffered saline (PBS) and fixed with 10% formaldehyde for 10 min. The fixed cells were washed three times with distilled water and stained with ORO solution (stock solution, 3 mg/mL in isopropanol; working solution, 60% ORO stock solution and 40% distilled water) for 30 min at room temperature. The cells were rinsed three times with distilled water. Finally, the ORO dye retained in the cells was eluted with isopropanol and quantified by measuring its absorbance at 500 nm.

### 2.5. Western Blot Analysis

To measure the expression of lipid accumulation-related proteins, western blot analysis was performed. HepG2 cells were washed with cold PBS and then collected by centrifugation. The washed cell pellets were lysed in a cell lysis buffer (iNtRON Biotech, Seongnam, Korea), according to the manufacturer's instructions. The protein concentration was determined using Take 3™ Multi-Volume Plate model in an Epoch plate reader (BioTek Instruments, Inc.) and calculated by Gen5 software (BioTek Instruments, Inc.). Proteins were separated by 10% sodium dodecyl sulfate- (SDS-) polyacrylamide gel and transferred onto nitrocellulose membranes. The membranes were blocked in a blocking solution (5% skim milk in Tris-buffered saline/Tween 20 [TBST]) for 1 h at room temperature. They were incubated for 3 h with polyclonal antibodies against p-AMPK, p-ACC, AMPK, ACC, FAS, SREBP-1c, and monoclonal anti-*β*-actin antibodies (1 : 1000 each) in TBST containing 5% skim milk. The membranes were then washed three times with TBST and incubated with horseradish-conjugated secondary antibody (1 : 1000) for 1 h at room temperature. The membranes were again washed three times with TBST. The specific protein bands were visualized on an X-ray film activated by chemiluminescence using ECL detection reagents. The autoradiograms were quantified by optical densitometry using ImageJ software (NIH, Washington, DC, USA).

### 2.6. Statistical Analysis

Data were expressed as the means ± standard errors (SE) of at least three independent experiments. Statistical analysis was performed by Duncan's test using SAS version 9.4 (SAS Institute, Inc., Cary, NC, USA). A *p* value < 0.05 was considered statistically significant.

## 3. Results and Discussion

### 3.1. Effects of Nobiletin on Cytotoxicity and Lipid Accumulation

**Figure 2:**
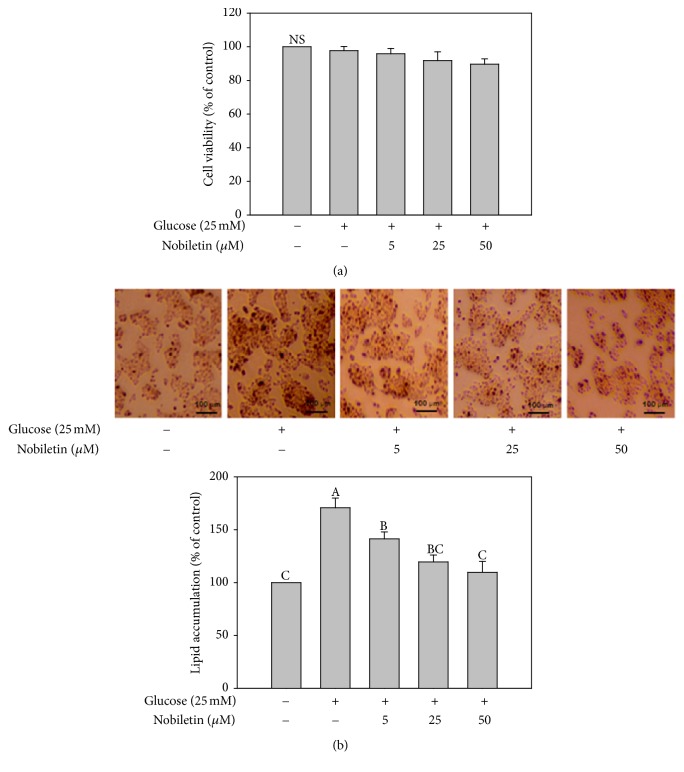
Effects of nobiletin on cytotoxicity (a) and lipid accumulation (b) in HepG2 cells. HepG2 cells were treated with high glucose (25 mM) and different concentrations of nobiletin for 24 h. Values are expressed as the means ± standard errors (*n* = 3). Different letters indicate a significant difference at *p* < 0.05. NS: nonsignificant difference between the control and all treatments (*p* < 0.05).

MTT assay was used to determine the effects of nobiletin on the viability of HepG2 cells. Cells were incubated in the presence or absence of nobiletin in DMEM containing high concentration of glucose (25 mM) for 24 h. As shown in Figure 2(a), no cytotoxicity was observed at any of the concentrations examined (5, 25, and 50 *μ*M). These results suggested that nobiletin did not affect the viability of HepG2 cells.

To examine the effects of nobiletin on lipid accumulation, HepG2 cells were cultured in DMEM containing high concentration of glucose (25 mM) and various concentrations of nobiletin (5, 25, and 50 *μ*M) for 24 h. ORO staining was performed to measure the total lipid accumulation in HepG2 cells. Nobiletin significantly reduced high glucose-induced intracellular lipid accumulation (Figure 2(b)). In line with these findings, a previous study showed that nobiletin suppressed lipid accumulation in 3T3-L1 cells [21]. In addition, nobiletin attenuated the activity of glycerol 3-phosphate dehydrogenase (GPDH) [21], which plays an important role in the conversion of glycerol to triglycerides [25]. The results of the present study showed that nobiletin exhibited inhibitory effects against lipid accumulation in HepG2 cells.

### 3.2. Effects of Nobiletin on SREBP-1c and FAS Expression

**Figure 3:**
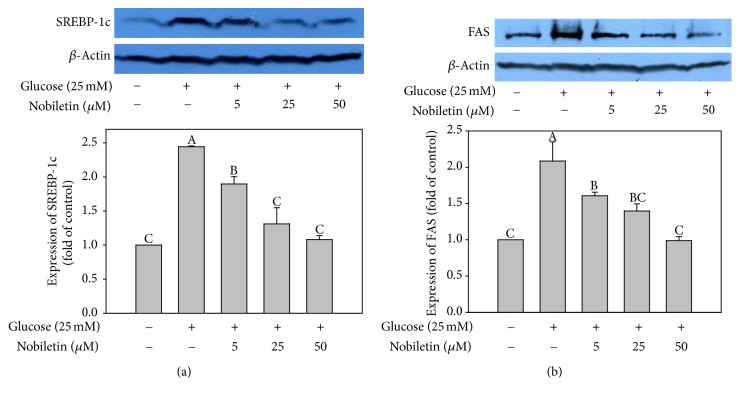
Effects of nobiletin on SREBP-1c (a) and FAS (b) expression in HepG2 cells. HepG2 cells were treated with high glucose (25 mM) and different concentrations of nobiletin for 24 h. Values are expressed as the means ± standard errors (*n* = 3). Different letters indicate a significant difference at *p* < 0.05. Blots are representative of at least three independent experiments.

Nobiletin treatment decreased the protein expression of SREBP-1c (Figure 3(a)) and FAS (Figure 3(b)) in high glucose-stimulated HepG2 cells in a concentration-dependent manner. The regulation of lipogenic gene expression by insulin and fatty acids is mainly mediated by transcription factors, such as SREBPs [26]. SREBP-1c, a glucose-dependent transcription factor, plays a crucial role in the regulation of the expression of lipogenic genes, such as FAS, in the liver [27]. Overexpression of genes that activate lipogenesis, such as SREBP-1, FAS, and ACC, has been observed in steatotic livers of humans and obese mice [28, 29]. These results indicated that nobiletin suppressed lipid accumulation via inhibition of the expression of lipogenic proteins, such as SREBP-1c and FAS, in HepG2 cells. In addition, these findings suggested that SREBP-1c and FAS might be the targets of nobiletin with regard to its hypolipidemic effects in hepatocytes.

### 3.3. Effects of Nobiletin on Phosphorylation of AMPK and ACC

**Figure 4:**
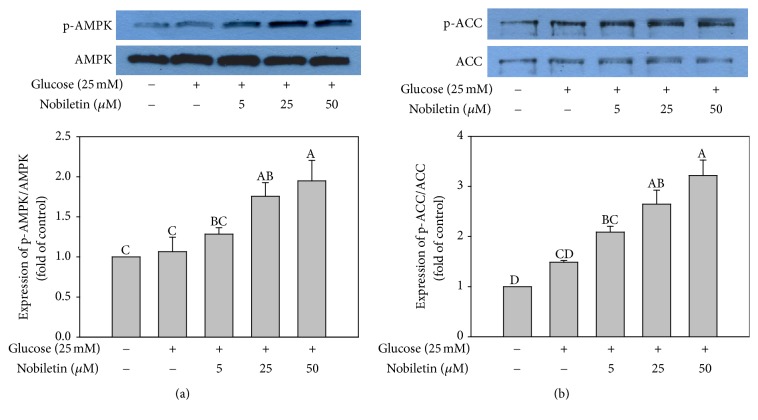
Effects of nobiletin on AMPK (a) and ACC (b) phosphorylation in HepG2 cells. HepG2 cells were treated with high glucose (25 mM) and different concentrations of nobiletin for 24 h. Values are expressed as the means ± standard errors (*n* = 3). Different letters indicate a significant difference at *p* < 0.05. Blots are representative of at least three independent experiments.

To investigate the effect on the phosphorylation of AMPK and ACC by high glucose conditions, HepG2 cells were incubated in serum-free DMEM containing high concentration of glucose (25 mM) in the absence or presence of nobiletin (5, 25, and 50 *μ*M) for 24 h. Nobiletin treatment significantly increased the expression of p-AMPK and p-ACC in a concentration-dependent manner (Figures 4(a) and 4(b)). It has been shown that AMPK modulates several key lipid metabolism-related transcription factors, including SREBP-1c [30]. The activation of AMPK suppresses SREBP-1c expression [31] and inhibits its proteolytic processing and transcriptional activity [30]. 5-Aminoimidazole-4-carboxamide-riboside (AICAR), an AMPK activator, was shown to inhibit adipocyte differentiation and block the expression of late adipogenic markers, such as peroxisome proliferator-activated receptor gamma (PPAR*γ*) and CCAAT-enhancer-binding protein alpha (C/EBP*α*) in adipocytes [32]. In a previous study, once the suppression of AMPK signaling was compensated, the increase in lipid accumulation was obviously abolished [33]. These results showed that activation of AMPK by nobiletin might, in part, be involved in the inhibition of lipid accumulation in HepG2 cells.

### 3.4. Involvement of AMPK in the Inhibitory Effects of Nobiletin on Lipid Accumulation

**Figure 5:**
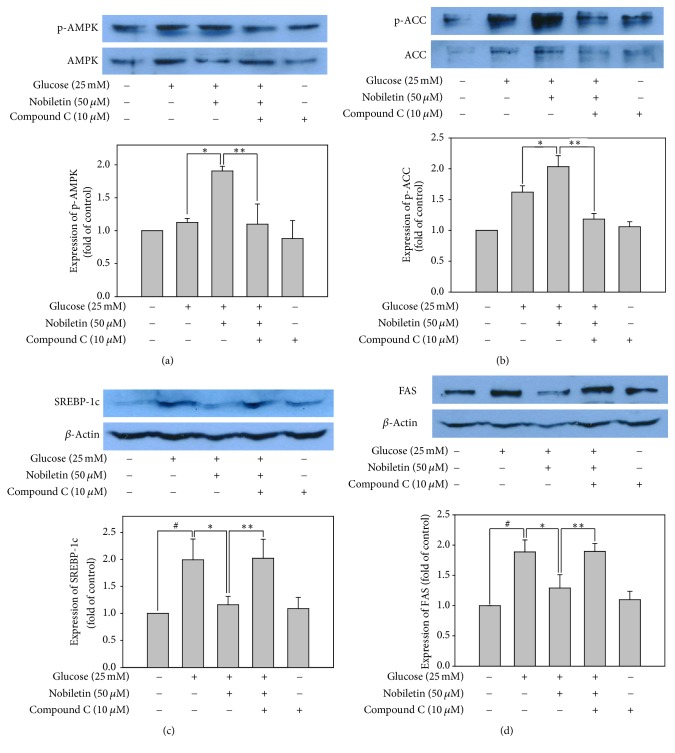
Involvement of AMPK in the inhibitory effects of nobiletin on lipid accumulation. The protein expression levels of phosphorylated AMPK (a), phosphorylated ACC (b), SREBP-1c (c), and FAS (d). Values are expressed as the means ± standard errors (*n* = 3). ^*∗*^*p* < 0.05 versus high glucose-treated cells; ^#^*p* < 0.05 versus control; ^*∗∗*^*p* < 0.05 versus high glucose plus 50 *μ*M nobiletin-treated cells. Blots are representative of at least three independent experiments.

To confirm the role of AMPK in the regulation of lipid metabolic gene expression, HepG2 cells were treated with 10 *μ*M compound C, an AMPK inhibitor, in serum-free medium for 1 h, and then incubated with or without 50 *μ*M nobiletin in DMEM containing high concentration of glucose (25 mM) for an additional 24 h. The increase in p-AMPK and p-ACC expression by nobiletin was completely abolished by compound C pretreatment in HepG2 cells (Figures 5(a) and 5(b)). These results indicated that nobiletin induced ACC phosphorylation by activating AMPK signaling pathway in HepG2 cells.

To elucidate whether inhibition of several key lipid metabolism-related transcription factors by nobiletin might be regulated by AMPK activation, the protein expression of SREBP-1c and its target lipogenic enzymes, such as FAS, was evaluated. Although nobiletin significantly reduced the expression of SREBP-1c and FAS in high glucose-treated HepG2 cells, compound C pretreatment abolished these inhibitory effects of nobiletin (Figures 5(c) and 5(d)). AMPK activation decreases lipogenesis by inhibiting SREBP-1c activation, thereby downregulating ACC and FAS expression [14]. AMPK directly phosphorylates SREBP-1c and SREBP-2. It was shown to stimulate Ser372 phosphorylation, suppress SREBP-1c cleavage and nuclear translocation, and repress SREBP-1c target gene expression in hepatocytes exposed to high glucose concentrations, resulting in reduced lipogenesis and lipid accumulation [30]. These results suggested that nobiletin could inhibit de novo lipogenesis via downregulation of lipogenic gene transcription in HepG2 cells by activating the AMPK signaling pathway.

**Figure 6:**
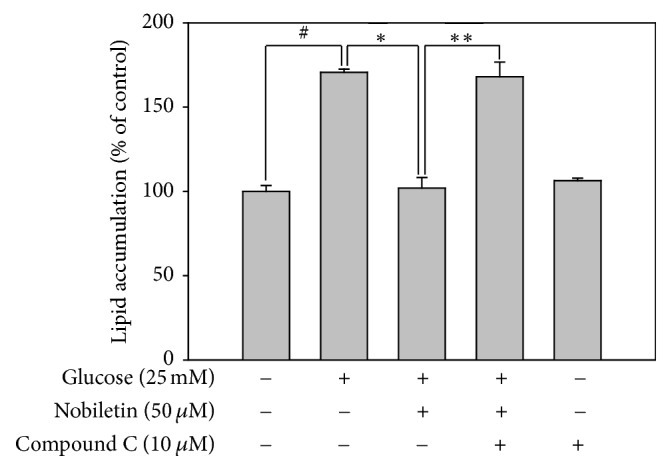
Involvement of AMPK pathway in the inhibitory effects of nobiletin and compound C, an AMPK inhibitor, on lipid accumulation in HepG2 cells. Quantification of accumulated lipids was measured by Oil red O staining. Values are expressed as the means ± standard errors (*n* = 3). ^#^*p* < 0.05 versus the control cells, ^*∗*^*p* < 0.05 versus high glucose-treated cells, and ^*∗∗*^*p* < 0.05 versus high glucose plus 50 *μ*M nobiletin-treated cells.

In addition, the effects of AMPK on the regulation of intracellular lipid accumulation were investigated in HepG2 cells. Total lipid content was measured by ORO staining. Lipid accumulation significantly decreased in high glucose-stimulated HepG2 cells treated with nobiletin; however, compound C increased lipid accumulation in HepG2 cells (Figure 6). A previous study showed that nobiletin markedly inhibited adipogenesis of preadipocytes in mature adipocytes by blocking the expression of the adipogenic transcription factors, such as PPAR*γ* and C/EBP*α* via activation of AMPK signaling pathway [21]. Moreover, various natural compounds, such as gomisin N and genistein, were shown to exert protective effects against hepatic steatosis by inhibiting lipogenesis and stimulating fatty acid oxidation through AMPK activation* in vivo* [34, 35]. Taken together, these results showed that nobiletin reduced total lipid content by activating AMPK signaling pathway in HepG2 cells.

## 4. Conclusions

In conclusion, we investigated the molecular mechanisms, by which nobiletin attenuated intracellular lipid accumulation in HepG2 cells. Nobiletin decreased SREBP-1c and FAS expression via activation of AMPK signaling pathway. To the best of our knowledge, this is the first study showing that AMPK activation by nobiletin attenuated lipid accumulation in HepG2 cells. Collectively, these results suggested that nobiletin might have been a potential functional food ingredient for preventing liver diseases, such as hyperlipidemia and hepatic steatosis.
